# Somatic mutation landscape of a meningioma and its pulmonary metastasis

**DOI:** 10.1186/s40880-018-0291-2

**Published:** 2018-05-04

**Authors:** Yaran Du, Ting Lu, Song Huang, Fangfang Ren, Gang Cui, Jian Chen

**Affiliations:** 1 Institute of Functional Nano and Soft Materials (FUNSOM) & Collaborative Innovation Center of Suzhou Nano Science and Technology, Soochow University, Suzhou, 215123 P. R. China; 2 Department of Neurosurgery, First Affiliated Hospital of Soochow University, Suzhou, 215006 P. R. China; 3 National Institute of Biological Sciences, Beijing, 102206 P. R. China; 4 Department of Biochemistry and Molecular Biology, Soochow University Medical College, Suzhou, 215123 P. R. China

**Keywords:** Meningiomas, Extracranial metastasis, Whole exome sequencing, Neurofibromin 2

## Abstract

**Background:**

Extracranial metastasis (ENM) of meningiomas is extremely rare, and typically occurs several years after a primary tumor is diagnosed. However, the genetic changes underlying ENM events have not yet been investigated.

**Case presentation:**

A 58-year-old male patient was sent to the emergency room of our hospital because of a sudden fall. Magnetic resonance imaging detected a mass at the right frontal sagittal sinus. He underwent tumor resection and recovered well, but post-operative computed tomography revealed three lumps on the right side of his chest. Thoracic surgery was performed to remove two of the lumps. Pathological findings revealed that the brain and lung tumors were grade I meningiomas. The patient received no additional radiation or chemotherapy post-surgery, and there was no sign of tumor recurrence in the brain or progression of the remaining lump in the chest 1 year after surgery. We performed whole exome sequencing of the patient’s blood, primary brain tumor, and lung metastatic tumor tissues to identify somatic genetic alterations that had occurred during ENM. This revealed that a frameshift deletion of the neurofibromin 2 gene likely drove formation of the meningioma. Surprisingly, we found that the brain tumor was relatively homogeneous and contained only one dominant clone; both the pulmonary metastasis and the original brain tumor were derived from the same clone, and no obvious additional driver mutations were detected in the metastatic tumor.

**Conclusion:**

Although ENM of meningiomas is very rare, brain tumor cells appear to be more adaptable to tissue microenvironments outside of the central nervous system than was commonly thought.

## Background

Meningiomas are primary central nervous system (CNS) tumors that originate from the arachnoid cap cells surrounding the brain [1]. They are the most common primary CNS tumors in the US [2] and the second most common in China [3]. The vast majority of meningiomas are considered benign and can be treated by surgical resection and adjuvant radiation therapy. As with other CNS tumors such as gliomas, extracranial metastasis (ENM) of meningiomas is extremely rare, occurring in only 0.1–0.2% of patients [4, 5].

Next-generation sequencing techniques have enabled characterization of the genomic landscape of primary meningiomas, including the discovery of several driver mutations in genes including NF2, TRAF7, KLF4, AKT1, and SMO [6–8]. However, to our knowledge, the mutation spectrum of metastatic meningioma has not previously been reported. Here, we present a patient with pulmonary metastasis of meningioma that was identified at the same time as diagnosis of a primary brain tumor. To explore the cause of ENM, we performed whole exome sequencing (WES) to reveal the somatic landscape of the primary brain tumor and its pulmonary metastasis.

## Case presentation

**Fig. 1:**
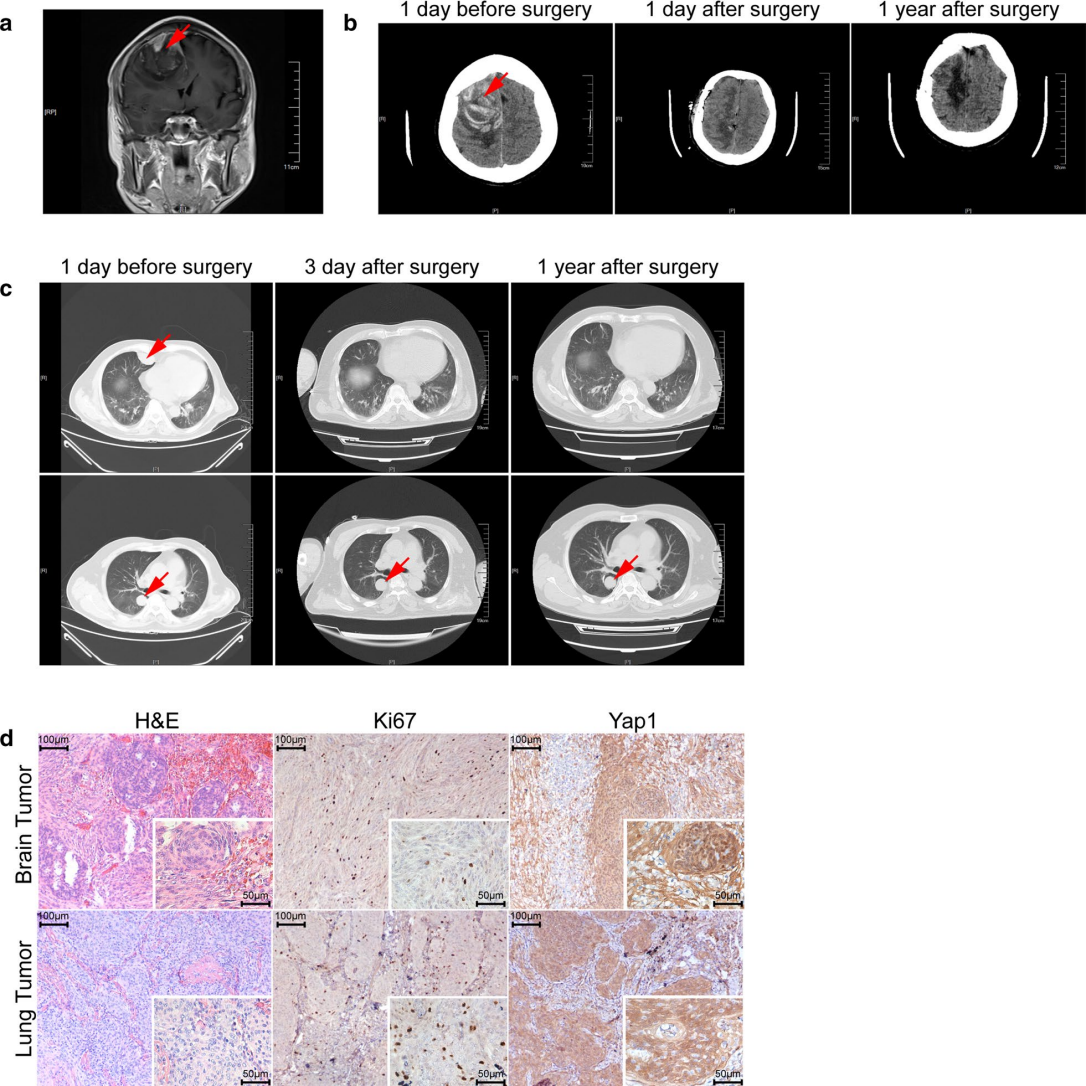
Imaging and histology of the primary brain meningioma and its pulmonary metastasis. **a** Coronal contrast-enhanced T1-weighted MRI imaging of the brain. **b** CT scan images of the brain before, post-brain surgery, and 1 year after surgery. **c** Axial CT scan images of the lung before, post-chest surgery, and 1 year after surgery. **d** H&E, Ki67, and Yap1 staining of the original brain meningioma (upper panels) and its pulmonary metastasis (lower panels)

A 58-year-old male patient who experienced a sudden fall was sent to the emergency room of the First Affiliated Hospital of Soochow University with speech difficulties and a lack of movement in his left limbs. On Aug. 26, 2016, a computed tomography (CT) scan of his head showed a hematoma in the upper part of the right forehead, and coronal contrast-enhanced T1-weighted magnetic resonance imaging (MRI) suggested a tumor lesion at the right frontal sagittal sinus (Fig. 1a, b).

The tumor mass was surgically removed and the patient recovered well. However, 3 days after the operation, a CT scan of his brain and chest revealed three lumps on the right side of the chest (Fig. 1c). Twenty-three days after the first operation, the patient underwent thoracic surgery to remove two lumps from the lung parenchyma. The third lump was not removed because it was very closely associated with the aorta and bronchi.

Post-operative pathology showed that both the intracranial tumor and lung tumors were grade I meningiomas with similar marker expression (epithelial membrane antigen negative, S100 protein negative, somatostatin receptor 2 positive, vimentin positive, signal transducer and activator of transcription 6 negative, smooth muscle actin negative, CD34a negative, cytokeratin AE1/AE3 negative and Ki67 3–5% positive). This diagnosis was independently confirmed by Huashan Hospital Fudan University (Fig. 1d). The patient received no additional radiation or chemotherapy post-surgery. At the 1-year follow-up visit, he was able to care for himself, and a further CT scan suggested no recurrence of the intracranial tumor and no progression of the remaining lump in the right chest (Fig. 1b, c).

**Fig. 2:**
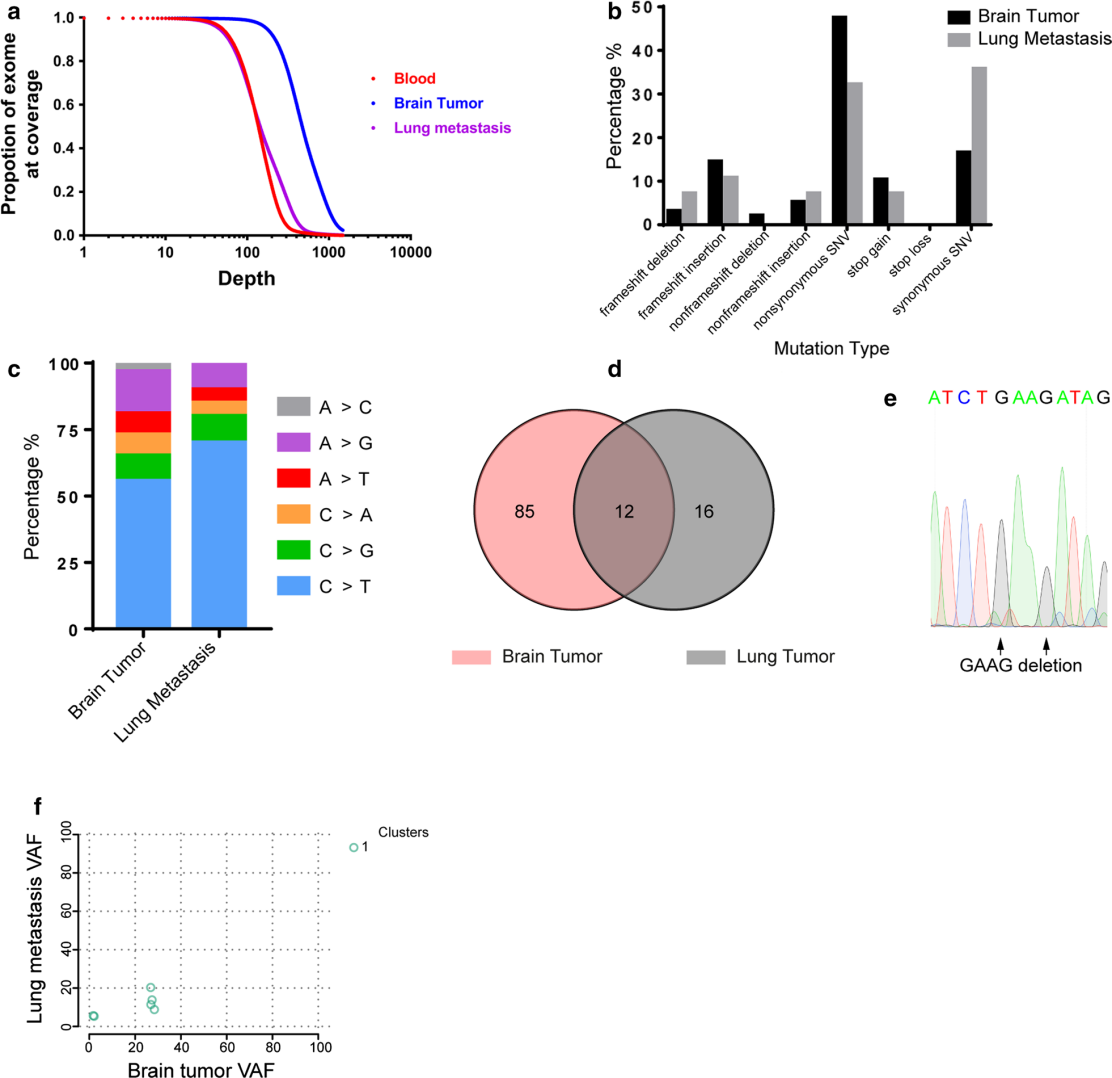
Summary of the whole exome sequencing results of the primary meningioma and its pulmonary metastasis. **a** Sequencing depth and coverage summary of the tumor and blood samples. **b**, **c** Frequency of somatic mutation types and substitution types. **d** Venn diagram of total somatic mutations in the original meningioma and its lung metastasis. **e** Sanger sequencing result verifying the *NF2* frameshift deletion region. **f** Two-dimensional analysis of the variant allele frequency in the primary brain tumor and lung metastasis samples

Extracranial metastases of meningiomas are extremely rare, so we performed WES of the patient using paraffin-embedded tumor tissues and blood samples to obtain an overview of the somatic mutation landscape. Blood and lung tumor tissue were sequenced at a depth of around 100×, while the brain tumor was sequenced at a depth of more than 500× to identify possible genetic heterogeneity that may be associated with metastasis (Fig. 2a).

WES revealed 97 and 28 somatic mutations in the brain tumor and lung metastasis, respectively, that passed the MuTect2 [9] filter with an allele frequency > 0.01 (Fig. 2b–d, Table 1). Twelve mutations (including five synonymous mutations) were shared by the original tumor and its metastasis (Fig. 2d). Among all mutations, the only known driver mutation causative of meningiomas was a frameshift deletion of NF2, which was present in both brain and lung tumors. We validated this mutation by Sanger sequencing (Fig. 2e). No other driver mutations from Intogen’s list of ~ 500 pan-cancer driver genes [10] were shared by the two tumors.

**Table 1:** Potential driver mutations and shared mutations between brain tumor and lung metastasis

Mutation	AA change	Exonic function	Brain AF	Lung AF	Intogen^a^	REVEL^b^	MCAP^c^
NF2	L125fs	Frameshift deletion	0.29	0.16	TIER-1		
YBX3	P164fs	Frameshift deletion	0.28	0.088			
SETD1B	S1366S	Synonymous SNV	0.28	0.20	Passenger		
CDT1	G164D	Nonsynonymous SNV	0.27	0.14	Not-protein-affecting	0.037	0.011
OR5AU1	D71V	Nonsynonymous SNV	0.27	0.11	Not-protein-affecting	0.20	0.011
NLGN2	H485D	Nonsynonymous SNV	0.27	0.20	Passenger	0.74	0.12
MPI	R272R	Synonymous SNV	0.27	0.12	Passenger		
DGKZ	A885A	Synonymous SNV	0.072	0.064	Passenger		
KRT86	A334A	Synonymous SNV	0.051	0.057	Passenger		
ANKS4B	F234F	Synonymous SNV	0.021	0.085	Passenger		
VPS13B	T3820 delinsMFS	Nonframeshift insertion	0.021	0.053	Passenger		
ANKS4B	F234fs	Frameshift insertion	0.017	0.056	Passenger		
PTPRC	R183fs	Frameshift insertion	0.014	ND	TIER-2		
TXNIP	A275fs	Frameshift insertion	0.013	ND	TIER-1		

*AF* allele frequency, *ND* not detected

^a^Potential of gene function categorized by Intogen

^b^REVEL score predicting functional impact of amino acid change to the protein

^c^MCAP score predicting functional impact of amino acid change to the protein

NF2 mutations are the most frequent in meningiomas, and loss of NF2 has been confirmed to cause meningioma in a mouse model [11]. Loss of NF2 protein also leads to the elevation of Yap1, a downstream effector protein [12], and we visualized increased Yap1 using immunohistochemistry in both the primary brain tumor and its lung metastasis (Fig. 1d). Among the other genes carrying nonsynonymous mutations that were shared between the brain and lung tumors (Table 1), the functions of YBX3 and CDT1 have previously been studied in different types of cancers [13, 14]. and YBX3 protein was reported to inhibit intestinal cell differentiation and promote epithelial cell proliferation [14, 15]. CDT1 is required to initiate DNA replication, while CDT1 overexpression promoted re-replication and malignant behavior [13, 16].

Our patient harbored a frameshift mutation of YBX3 and a CDT1 G164D mutation in both tumors. Since YBX3′s mRNA level in brain is very low [17] and it’s known to play an oncogenic role in cancers, the frameshift mutation of YBX3 in the patient is less likely to drive tumor formation. Although effect of G164D mutation on the CDT1 protein is unknown. We used REVEL [18] and M-CAP [19] algorithms to predict the consequence of the mutation, and found it to have a minimal impact on protein function (Table 1). The only known driver mutation detected by MuTect2 that was unique to the lung tumor was a G to A substitution in CHD8 that results in an R1303Q change in the protein sequence. However, the frequency of this mutation is low, and it failed to pass the filter of two additional caller algorithms, Strelka [20] and Varscan2 [21].

Genomic studies from other solid tumors suggested that tumors contain multiple subpopulations (clones) with distinct genomic profiles. These clones may grow, evolve, and regress during disease progression or treatment. Thus, tumors from one metastatic site usually evolve from one subclone of the original tumor to contain additional mutations [22]. It has also been established that increased intratumoral heterogeneity is associated with tumor recurrence in meningiomas [23]. To explore the heterogeneity of the original brain tumor and its relationship with pulmonary metastasis, we used the sciClone R package [24] to evaluate mutation data. Surprisingly, only one dominant clone was found in the brain tumor, and it was also shared by the metastatic tumor (Fig. 2f).

The immune system prevents extracranial metastasis of brain tumors such as glioblastomas (GBMs) [25] in a response involving MHC-I proteins [26]. We genotyped the type I human leukocyte antigen (HLA) of the patient using OptiType [27] and HLA-HD algorithms [28] but did not identify any mutations in HLA type I genes.

## Discussion and conclusions

The metastatic potential of tumors usually increases with their grades. Because meningiomas are mostly low-grade benign tumors, their ENM is particularly rare and only a small number of meningioma ENM cases have been described in the literature [5, 29]. However, ENM has been observed in both high-grade and low-grade meningioma cases [5], which probably results from the invasive action of meningioma cells, regardless of tumor grade. For example, the pulmonary metastasis presented here is from a grade I meningioma. Interestingly, for both gliomas and meningiomas, the most frequent organs involved in ENM are the lung and pleural cavity (60%) [4, 5].

As a result of its rarity, there are currently no markers available to predict ENM of CNS tumors, and no standard management regimes for related diseases. Various hypotheses have been developed to explain the rarity of ENM events, most of which attribute the dearth of events to the unique anatomical structure and microenvironment of the brain [30, 31]. First, it is difficult to compensate for the space-occupying lesion in the brain so the outcome of primary malignant brain tumors is often very poor, limiting the time to develop ENM. Indeed, it has been noted that the overall survival of patients who develop ENM in GBM is significantly longer than in GBM patients without ENM [4]. However, this is less likely to explain the low incidence of ENM events in meningiomas which have a far better outcome compared with GBM.

Second, cell dissemination from brain tumors is more difficult than dissemination from tumors in many peripheral organs. The blood–brain barrier likely plays a role in hindering brain tumor cell metastasis through the hematogeneous pathway. Thus, most (> 90%) ENM cases reported have occurred after surgery or shunting [30], two processes that facilitate tumor cell access to extra-meningeal vessels and lymphatic channels. However, it was recently reported that circulating tumor cells were found in 20.6% of GBM patients [32], suggesting that some enter the circulation at a relatively high frequency in the absence of surgery. Other mechanisms must also contribute to the low incidence of ENM.

Third, the distinct brain microenvironment means that brain tumor cells are unlikely to grow outside of the CNS. Metastatic tumor cells are thought to be selected for their ability to invade connective tissues, which contain large amounts of collagen and fibronectin. However, connective tissue is absent from the brain, and the major component of brain extracellular matrix is hypertonic acid and other glycosaminoglycans [33].

Finally, immune surveillance plays an important part in suppressing ENM. The brain was originally considered to be immune privileged, and it was only very recently that lymphatic vessels were discovered in the meninges and shown to drain out through deep cervical lymph nodes [34]. Nevertheless, the immune response in the CNS differs from that of peripheral sites. This difference is clearly seen in rodent tumor transplantation experiments. For example, F98 rat glioma cells were able to grow in syngeneic rat brains but failed to thrive in 97% of cases when injected subcutaneously [25]. Similarly, studies using allograft assays showed that the DBT glioma cell line survived in the brain but not in other organ sites [35].

In the present case, multiple lung metastases were found at the same time that the primary brain tumor was diagnosed. Thus, the possibility that the ENM occurred through surgically-facilitated tumor cell spreading can be excluded. During surgery, we found that the primary brain tumor had invaded the skull base, suggesting that tumor cells may have entered the circulation through meningeal vessels.

To our knowledge, this is the first time that the somatic landscape of an ENM tumor and its paired brain tumor has been characterized. The present meningioma appears to have been driven by inactivation of NF2 and the subsequent accumulation and activation of the Yap1 protein (Fig. 1d). The inactivation of NF2 accounts for around half of all meningiomas and more than 80% of high-grade meningiomas [6–8]. Moreover, deletion of the NF2 locus has previously been reported in pulmonary metastasis of meningiomas [36], but its relationship with ENM remains to be studied. Rather surprisingly, we were unable to find obvious additional mutations that may have contributed to the observed ENM events. This contrasts with the identification of additional genetic alterations in brain metastases from tumors of peripheral organs, especially in the PI3K/AKT/mTOR pathway [37].

A lack of somatic mutation events in primary tumors has been observed in some pediatric brain tumors (e.g. ependymomas), and epigenetic changes are also thought to be important for tumorigenesis [38]. Furthermore, no additional somatic mutations between the primary tumor and metastases were reported in other tumors such as liver metastases of colon cancer [39]. These observations suggest that tumor cells, even at a low grade, can adapt to a new environment without undergoing obvious genetic selection despite large differences in microenvironments between the original organ and its metastatic target sites. Further studies of gene expression and the epigenetic landscape in these cases may provide more clues about the formation of tumor metastases.

## Abbreviations

CNS: central nervous system
CT: computed tomography
ENM: extracranial metastasis
GBM: glioblastoma
MRI: magnetic resonance imaging
WES: whole exome sequencing
